# Stage-dependent dynamics of Apolipoprotein C3 across the spectrum of MASLD

**DOI:** 10.1371/journal.pone.0349666

**Published:** 2026-06-23

**Authors:** Eva Katharina Messer, Janett Fischer, Toni Herta, Albrecht Boehlig, Madlen Matz-Soja, Adrienn Tuennemann-Tarr, Susanne Gaul, Ulrich Laufs, Thomas Berg

**Affiliations:** 1 Division of Hepatology, Department of Medicine II, Leipzig University Medical Center, Leipzig, Germany; 2 Department of Hepatology and Gastroenterology, Charité-Universitätsmedizin Berlin, Campus Virchow-Klinikum (CVK) and Campus Charité Mitte (CCM), Berlin, Germany; 3 Berlin Institute of Health at Charité-Universitätsmedizin Berlin, BIH Biomedical Innovation Academy, BIH Charité Clinician Scientist Program, Berlin, Germany; 4 Clinic for Internal Medicine, Community Hospital Delitzsch, Delitzsch, Germany; 5 Clinic of Cardiology, Department of Medicine IV, Leipzig University Medical Center, Leipzig, Germany; 6 LeiCeM - Leipzig Center of Metabolism, Leipzig University, Leipzig, Germany; Medizinische Fakultat der RWTH Aachen, GERMANY

## Abstract

**Background:**

Metabolic dysfunction-associated steatotic liver disease (MASLD) is a systemic disorder closely linked to cardiometabolic risk and dysregulated lipid metabolism. Apolipoprotein C3 (ApoC3), a key regulator of triglyceride-rich lipoproteins and mediator of cardiovascular disease, has been implicated in hepatic steatosis. However, its stage-specific role across the MASLD spectrum remains incompletely defined.

**Methods:**

In this cohort study, serum ApoC3 concentrations were quantified in 197 patients with MASLD across different disease stages (non-fibrotic MASLD, fibrosis, cirrhosis, and cirrhosis with hepatocellular carcinoma (HCC)) and compared with 204 blood donor controls. Associations with metabolic parameters, non-invasive fibrosis markers, histology, cardiometabolic comorbidities, and genetic risk variants were analyzed.

**Results:**

ApoC3 levels exhibited a stage-dependent pattern, with significantly lower concentrations in cirrhotic MASLD and HCC compared to non-fibrotic MASLD and controls (median 18.7 vs. 26.6 vs. 27.4 mg/dL, p < 0.05). Lower ApoC3 levels were associated with markers of advanced liver disease, including higher bilirubin, liver stiffness, Fibrosis-4 index, and NAFLD fibrosis score. In contrast, higher ApoC3 levels were observed in early-stage MASLD and were associated with cardiometabolic comorbidities and dyslipidemia. ApoC3 correlated positively with triglycerides (r = 0.485, p < 0.001) and controlled attenuation parameter values (r = 0.274, p = 0.004), and was highest in patients with severe steatosis (S3) and increased inflammatory activity (NAS ≥ 5). Diagnostic performance for cirrhosis was modest (AUC 0.642) and for HCC 0.673.

**Conclusion:**

ApoC3 demonstrates a biphasic, stage-dependent profile in MASLD, with elevated levels reflecting hepatic steatosis, and metabolic dysfunction in early disease, and declining levels likely indicating impaired hepatic function in advanced stages. These findings position ApoC3 as a link between hepatic lipid accumulation and cardiovascular risk rather than a linear marker of disease severity. Its clinical utility may lie in integrative, multi-parameter models rather than as a standalone biomarker.

## Introduction

Metabolic dysfunction-associated steatotic liver disease (MASLD) is a significant global public health issue [1]. MASLD refers to a spectrum of liver disorders, ranging from simple hepatic steatosis to metabolic dysfunction-associated steatohepatitis (MASH), which carries a higher risk of progression to fibrosis, cirrhosis, and hepatocellular carcinoma (HCC). MASLD is increasingly recognized as a systemic disorder, in which hepatic pathology is closely linked to broader metabolic derangements, including dyslipidemia, insulin resistance, and chronic inflammation, leading to increased risk of cardiovascular and liver-related death [2,3].

As the diagnosis of MASH moves from liver biopsy to non-invasive methods, reliable biomarkers are crucial. One such potential biomarker is apolipoprotein C3 (ApoC3) [4,5], a liver-derived glycoprotein that plays a pivotal role in the regulation of triglyceride-rich lipoprotein metabolism by inhibiting lipoprotein lipase and hepatic uptake of triglyceride-rich particles [5–8]. Elevated ApoC3 levels are strongly associated with hypertriglyceridemia, atherogenic dyslipidemia, and increased cardiovascular risk [9–12]. Importantly, insulin resistance — a central pathophysiological driver of MASLD — influences ApoC3 expression in hepatocytes. Hyperinsulinemia and impaired insulin signaling promote increased ApoC3 secretion, linking metabolic dysfunction to altered lipid handling at both hepatic and systemic levels [13–17].

Given this mechanistic connection, ApoC3 may serve as a stage-dependent biomarker in MASLD, reflecting not only hepatic lipid accumulation but also systemic metabolic stress. While previous studies have examined ApoC3 primarily in the context of cardiovascular disease and dyslipidemia, its role across the spectrum of MASLD, particularly in relation of disease progression, remains incompletely understood.

Therefore, this study aimed to quantify serum ApoC3 concentrations in a well-characterized MASLD cohort across different disease stages and to examine their associations with metabolic, biochemical, and non-invasive markers of liver pathology. By integrating insights from hepatology and cardiometabolic medicine, our work seeks to elucidate the complex interplay between cardiovascular risk factors, lipid metabolism, and progressive liver dysfunction in MASLD.

## Materials and methods

### Ethical statement

The study was approved by the Ethics Committee of the University of Leipzig (vote no. 372/19-ek) and conducted in accordance with the Declaration of Helsinki (2013 revision) and ICH-GCP guidelines. Written informed consent was obtained from all participants. Authors had no access to information that could identify individuals during or after data collection.

### Study population

Patients with MASLD at various disease stages were retrospectively selected between January and April 2025 from the biobank of our hepatology outpatient unit which was recruited between 2020 and 2025. Eligible patients had hepatic steatosis on imaging, regardless of fibrosis stage. The diagnosis of MASLD was defined according to EASL criteria [18].

Exclusion criteria included significant alcohol intake (> 30 g/day for men, > 20 g/day for women; assessed via patient history), extrahepatic malignancies, benign liver tumors and alternative reasons for hepatic steatosis (e.g. Wilson’s disease or chronic viral hepatitis). Patients with cirrhotic MASLD and HCC were included as a distinct subgroup. HCC was diagnosed radiologically or histologically. The MASLD cohort was stratified into four groups: non-cirrhotic MASLD (no sign of fibrosis/cirrhosis), fibrosis, cirrhosis (without HCC), and cirrhosis with HCC. Fibrosis and cirrhosis were diagnosed via vibration-controlled transient elastography (VCTE) with values of 8−12 kPa for fibrosis and > 12 kPa for cirrhosis, ultrasound, or imaging morphology. Fibrosis scores (NAFLD fibrosis score (NFS), Fibrosis-4 (FIB-4) index) and the FibroScan-AST (FAST) score were calculated to assess disease severity and detect active MASH (NAFLD activity score (NAS) ≥ 4, fibrosis stage ≥ F2) [19–21]. A control group of 204 Caucasian blood donors was included for comparison.

### Quantification of ApoC3

Serum samples were collected at initial presentation and stored at −20 °C. Serum levels of Apolipoprotein CIII (ApoC3) were quantified using a commercially available human ELISA kit (Abcam, #ab154131, (Cambridge, UK)) according to the manufacturer’s protocol. Reagents were equilibrated to room temperature prior to use. The standard curve ranged from 0 to 500 pg/ml, prepared by serial dilution in Diluent M. All samples and standards were run in duplicates using 50 µl per well. Plates were incubated for 2 hours at room temperature, washed five times with 200 µl wash buffer, followed by addition of biotinylated ApoC3 antibody (1:50 in Diluent M) and incubation for 1 hour. After washing, 50 µl of 1 × streptavidin-peroxidase conjugate was added and incubated for 30 minutes, followed by TMB substrate and stop solution. Absorbance was measured at 450 nm with 570 nm background correction. To ensure inter-assay consistency, each ELISA plate included 50% healthy control serum and 50% patient serum from MASLD cases. An internal plate control (serum standard) was used across all plates for normalization. All data points were within the linear range of the standard curve and are reported as normalized concentrations relative to the internal control.

### Genotyping

Both the MASLD and the control population were genotyped for established MASLD- and lipid- associated risk variants, including *PNPLA3* rs738409, *TM6SF2* rs58542926, *MBOAT7* rs641738, *GCKR* rs1260326, and *HSD17B13* rs72613567, as well as *APOC3* variants rs2854116 and rs2854117. Based on these loci, the polygenic risk score (PRS-5) was calculated to assess individual genetic predisposition for fibrosis progression and HCC among patients with MASLD [22].

### Liver histology

When available, liver biopsy results were included. All liver biopsies were performed in the context of clinical practice. Steatosis grade (S0-S3) and fibrosis stage (F0-F4) were assessed using standard criteria (percentage of hepatocellular fat and METAVIR scoring). Disease stage in MASLD was defined by fibrosis stage: intermediate (≥F2), advanced (≥F3), and cirrhosis (F4). Additionally, the NAS (0–8 points for steatosis, ballooning, and lobular inflammation) was calculated [23]. NAS scores of 0–2 were mostly non-diagnostic for MASH, scores of 3–4 were indeterminate, and scores ≥ 5 were generally diagnostic of MASH.

### Statistical analysis

Statistical analyses were performed using SPSS (Version 29, SPSS Inc., Chicago, IL, USA) and GraphPad Prism (Version 10, GraphPad Software, San Diego, CA, USA). Categorical variables are reported as frequencies (%) and continuous variables as median (range). Fisher’s exact test was used for categorical comparisons; Mann-Whitney U and Kruskal-Wallis tests with Dunn’s test post-hoc analysis for continuous variables, with a Bonferroni correction applied to account for multiple testing. Aligned Rank Transform (ART) ANOVA was used to analyze the interaction between groups. The diagnostic performance of ApoC3 was evaluated using receiver operating characteristic (ROC) analysis. The optimal cut-off was defined using Youden’s index [24], however, this threshold was derived for exploratory purposes and not intended for clinical decision-making. Corresponding sensitivity, specificity, positive predictive value (PPV), and negative predictive value (NPV) were calculated. Univariate and multivariate logistic regression analyses assessed associations between ApoC3 levels and liver disease status, with odds ratios (OR) and 95% confidence intervals (CI) reported. Spearman’s correlation was used to examine associations between ApoC3, laboratory parameters, and PRS-5. Analyses were performed using R (v4.4.2). Two-sided p-values <0.05 were considered significant.

## Results

### Population characteristics

A total of 197 MASLD patients at different disease stages and 204 blood donor controls were retrospectively analyzed (Table 1). Patients with cirrhosis and HCC were older (median 68 years, range 48–83) and predominantly male (87%) compared to those with cirrhosis without HCC (61 years, 49% male), fibrosis (57 years, 45% male), non-fibrotic MASLD (51 years, 38% male), and controls (57 years, 59% male). Among patients with HCC, only four presented without cirrhosis.

**Table 1:** Baseline characteristics of the study cohort.

Parameter	Non-fibrotic MASLD (n = 52)	Fibrosis (n = 22)	Cirrhosis (n = 76)	HCC (n = 47)	Controls (n = 204)	MASLD vs. Fibrosis*	MASLD vs. Cirrhosis*	MASLD vs. HCC*	Cirrhosis vs. HCC*
Male sex	20 (38%)	10 (50%)	37 (49%)	41 (87%)	120 (59%)	1.000	1.000	1.93x10^-06^	6.28x10^-05^
Age (years)	51 (21-71)	57 (30-78)	61 (26-83)	68 (48-83)	57 (18-78)	0.139	3.76x10^-07^	5.85x10^-16^	0.0008
BMI (kg/m^2^)	29.7 (18.4-45.9)	31.1 (19.7-43.9)	32.0 (20.2-64.3)	31.1 (17.2-42.6)	n/a	1.000	0.657	1.000	1.000
Diabetes	10 (19%)	11 (50%)	50 (66%)	36 (77%)	n/a	0.044	1.04x10^-06^	3.51x10^-08^	0.920
Arterial hypertension	26 (50%)	13 (59%)	47 (62%)	35 (74%)	n/a	1.000	0.824	0.056	0.688
Dyslipidemia	38 (73%)	16 (73%)	46 (61%)	19 (40%)	n/a	1.000	0.555	0.004	0.056
CHD	1 (2%)	2 (9%)	17 (22%)	10 (21%)	n/a	0.836	0.003	0.012	1.000
Previous heart attack	0	1 (5%)	3 (4%)	3 (6%)	n/a	1.000	1.000	0.424	1.000
Previous stroke	0	0	1 (1%)	3 (6%)	n/a	n/a	1.000	0.424	0.620
Child Pugh A B C			50 (66%) 23 (30%) 3 (4%)	34 (79%) 9 (21%) 0					0.203
Portal hypertension^#^			44 (58%)	17 (36%)					0.026
Treatment with cholesterol- lowering drugs	11 (21%)	2 (9%)	21 (28%)	9 (19%)	n/a	0.380	0.533	1.0000	0.388
CAP (db/m)	317 (139-401)	320 (208-400)	312 (100-400)	342 (100-393)	n/a	1.000	1.000	1.000	1.000
LSM (kPa)	5.2 (2.1-17.2)	9.0 (6.1-13.7)	27.6 (10.5-75.0)	14.6 (4.4-63.0)	n/a	0.008	7.42x10^-23^	0.008	0.172
FIB-4 Score	1.24 (0.24-6.00)	1.54 (0.44-8.93)	3.23 (0.92-12.85)	4.30 (1.01-25.12)	n/a	0.044	2.08x10^-14^	1.35x10^-15^	0.239
NFS	−2.50 (−6.22-3.29)	−0.96 (−4.21-4.28)	1.24 (−3.88-6.37)	1.78 (−4.57-7.47)	n/a	0.041	7.75x10^-13^	22.69x10^-14^	0.242
FAST-Score	0.36 (0.03-0.92)	0.59 (0.27-0.87)	0.79 (0.19-0.94)	0.77 (0.06-0.97)	n/a	0.094	1.50x10^-09^	0.315	0.998
ALT (U/L)	57 (3.6-205.2)	49.2 (22.8–186.6)	33.6 (7.2–263.4)	37.8 (12.6–117.6)	n/a	1.000	0.017	0.079	1.000
AST (U/L)	39.6 (15.6–175.2)	42.6 (22.2–222)	48 (13.8–309)	54.6 (22.8–153)	n/a	0.720	0.720	0.020	0.232
ALT/AST ratio	0.73 (0.22–7.50)	0.83 (0.22–8.41)	1.24 (0.49–4.26)	1.42 (0.46–7.58)	n/a	0.737	3.38x10^-7^	2.45x10^-08^	0.551
GGT (U/L)	64.2 (16.2–711)	80.4 (28.2–690)	117.6 (18.6–	232.8 (53.4–1614)	n/a	0.314	0.002	4.84x10^-09^	0.0007
AP (U/L)	84.6 (9.6–180)	89.4 (50.4–287.4)	111 (36–867.6)	160.8 (38.4–709.2)	n/a	0.421	0.003	1.08x10^-08^	0.004
Bilirubin (µmol/L)	9.2 (1.6-42.8)	9.4 (4.7-517.2)	15.8 (3.1-476.6)	13.7 (5.3-63.5)	n/a	0.768	1.21x10^-05^	0.003	0.768
Albumin (g/L)	47.6 (38.6-55.7)	45.1 (33.6-51.9)	41.8 (27.0-51.3	39.1 (30.4-47.5)	n/a	0.279	5.90x10^-09^	3.22x10^-11^	0.279
Platelets (x10^9^/L)	265 (141-448)	207 (101-404)	132 (42-437)	147 (44-639)	n/a	0.025	2.00x10^-14^	3.28x10^-10^	0.540
Leucocytes (x10^9^/L)	6.7 (3.9-12.6)	7.3 (3.6-11.8)	5.9 (1.8-16.9)	8.1 (1.4-15.6)	n/a	1.000	0.585	1.000	1.000
Total cholesterol (mmol/L)	5.37 (3.16-7.29)	5.61 (3.20-8.46)	4.90 (0.73-9.42)	4.83 (2.75-8.55)	n/a	0.885	0.587	0.587	0.885
LDL (mmol/L)	3.42 (1.69-5.64)	3.63 (1.51-7.04)	2.86 (0.21-7.96)	2.50 (1.36-6.55)	n/a	0.885	0.229	0.140	0.885
HDL (mmol/L)	1.26 (0.54-3.01)	1.20 (0.13-1.73)	1.26 (0.11-2.59)	1.23 (0.38-2.27)	n/a	1.000	1.000	1.000	1.000
Triglycerides (mmol/L)	1.92 (0.56-6.34)	2.35 (0.92-5.02)	1.46 (0.47-4.38)	1.45 (0.64-3.45)	n/a	0.819	0.113	0.119	0.819
CRP (mg/L)	1.74 (0.30-8.82)	4.41 (0.60-25.83)	3.61 (0.40-39.96)	7.14 (0.47-57.33)	n/a	0.095	0.0002	3.38x10^-08^	0.095

*p-value, ^#^defined as VCTE >15 kPa and platelets < 150x10^9^/1 [25] Values are presented as frequency (%) or median (range). ALT: alanine aminotransferase, AP: alkaline phosphatase, AST: aspartate aminotransferase, BMI: body mass index, CAP: controlled attenuation parameter, CHD: coronary heart disease, CRP: C-reactive protein, FAST Score: FibroScan-AST Score, FIB-4: fibrosis-4 score, GGT: gamma–glutamyl transpeptidase, HCC: hepatocellular carcinoma, HDL: high density lipoprotein, LDL: low-density lipoprotein, LSM: liver stiffness measurement, MASLD: metabolic dysfunction-associated liver disease, n/a: not applicable, NFS: non-alcoholic fatty liver disease fibrosis score. Statistics with Bonferroni correction.

### Correlation of ApoC3 with serum lipid parameters and non-invasive markers of MASLD progression

In the study cohort, serum ApoC3 concentrations showed moderate positive correlations with total cholesterol (r = 0.300, p = 2.98x10^-4^), low-density lipoprotein (LDL) cholesterol (r = 0.289, p = 4.19x10^-04^), and triglycerides (r = 0.485, p = 7.44x10^-10^). Patients with dyslipidemia showed higher serum ApoC3 levels compared to patients with a normal lipid profile (median 24.4 mg/dL [range 2.0–235.0] vs. 15.6 mg/dL 3.7–61.4], p = 0.0003).

Positive correlations were also observed with platelet count (r = 0.263, p = 2.61x10^-04^), serum albumin (r = 0.291, p = 6.86x10^-05^) and leucocyte count (r = 0.174, p = 0.017). CAP values were associated with higher serum ApoC3 concentrations (r = 0.274, p = 0.004, conducted in 111 patients).

**Fig 1:**
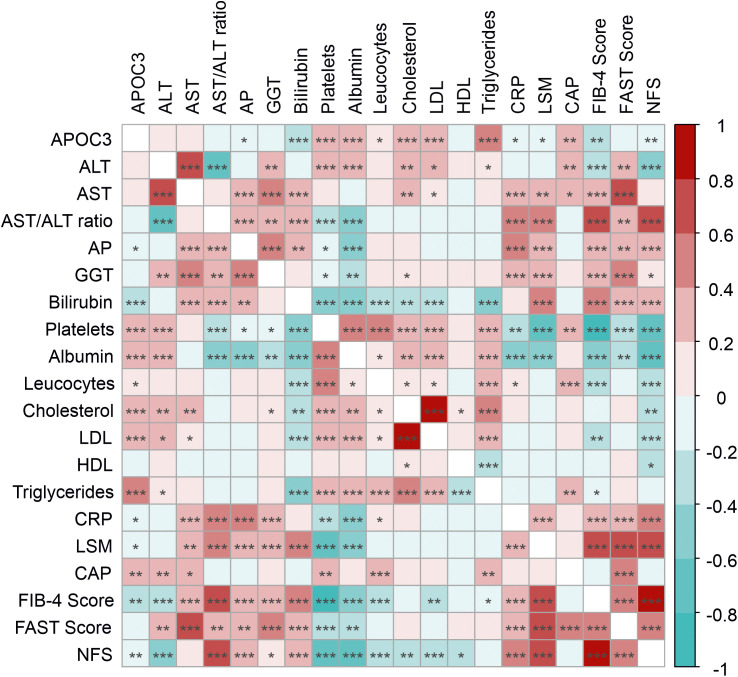
Correlation of serum apolipoprotein C3 (ApoC3) concentration with laboratory parameters, non-invasive measures for steatosis and fibrosis and MASLD scores for non-invasive assessment of liver disease. Statistics: Spearman’s correlation coefficient. Levels of significance: * p < 0.05, ** p < 0.01 and *** p < 0.001. ALT: alanine transaminase, AP: alkaline phosphatase, AST: aspartate transaminase, CAP: controlled attenuation parameter, CRP: C-reactive protein, FAST: FibroScan-AST score, FIB-4: fibrosis-4 score, GGT: gamma-glutamyl transferase, HDL: high-density lipoprotein, LDL: low-density lipoprotein, LSM: liver stiffness measurement, NFS: nonalcoholic fatty liver disease fibrosis score.

Lower serum ApoC3 concentrations were associated with higher bilirubin (r = −0.314, p = 1.07x10^-5^), alkaline phosphatase (r = −0.162, p = 0.026) and CRP (r = −0.177, p = 0.021), as well with increased LSM values (r = −0.193, p = 0.025, conducted in 136 patients), FIB-4 index (r = −0.201, p = 0.006) and NFS (r = −0.200, p = 0.009) (Fig 1, S1 and S2 Tables). Serum ApoC3 concentrations were not significantly associated with sex (p = 0.587), BMI (p = 0.078), the presence of diabetes (p = 0.198), arterial hypertension (p = 0.478), coronary heart disease (p = 0.254), or previous strokes (p = 0.336) and with portal hypertension (p = 0.251)

### Serum ApoC3 concentrations and histological signs of liver inflammation

**Fig 2:**
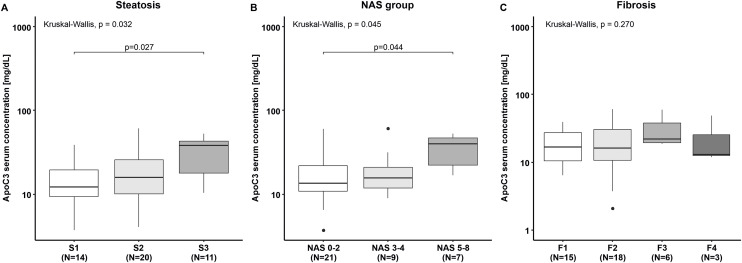
Serum apolipoprotein C3 (ApoC3) concentration in patients with MASLD and available liver biopsy according to the A) steatosis grade with S1: 5-33%, S2: 34-66%, S3: > 67% of fat within hepatocytes, B) NAFLD activity score (NAS) with NAS 0-2: no metabolic dysfunction-associated steatohepatitis (MASH), NAS 3-4: borderline NASH and NAS 5-8 histologic diagnosis of MASH, and C) fibrosis stage with F1: portal fibrosis without septa, F2: few septa, F3: numerous septa without cirrhosis, F4: cirrhosis. Box plots display the median value (-) and the range of the data set, as indicated by the whiskers. Statistics: Kruskal-Wallis-test (Dunn’s test post hoc).

Liver biopsy was performed in 53 MASLD patients at different disease stages. Characteristics of this subgroup are shown in S3 Table. Steatosis grading was available for 45 patients: 31% had S1, 44% S2, and 24% S3. Fibrosis stage was assessed in 42 cases (F1: 36%, F2: 43%, F3: 14%, F4: 7%), and NAS scores were available for 36 patients (0–2: 57%, 3–4: 24%, 5–8: 19%). ApoC3 levels correlated with both steatosis grade and NAS (Fig 2). Highest levels were seen in patients with S3 steatosis (38.1 mg/dL) compared to S2 (16.1 mg/dL) and S1 (12.1 mg/dL; p = 0.032). Similarly, ApoC3 was elevated in patients with NAS 5–8 (40.3 mg/dL) versus NAS 0–2 (13.5 mg/dL) and NAS 3–4 (15.6 mg/dL; p = 0.045). These findings suggest that ApoC3 is associated with both hepatic fat accumulation and inflammatory activity. No clear differences were observed across fibrosis stages, and interpretation in this cohort should be cautious due to the small number of patients with advanced fibrosis (only four patients with F4), which limits statistical power and may bias results toward more advanced disease.

### Serum ApoC3 concentrations in patients with cirrhosis with and without HCC

**Fig 3:**
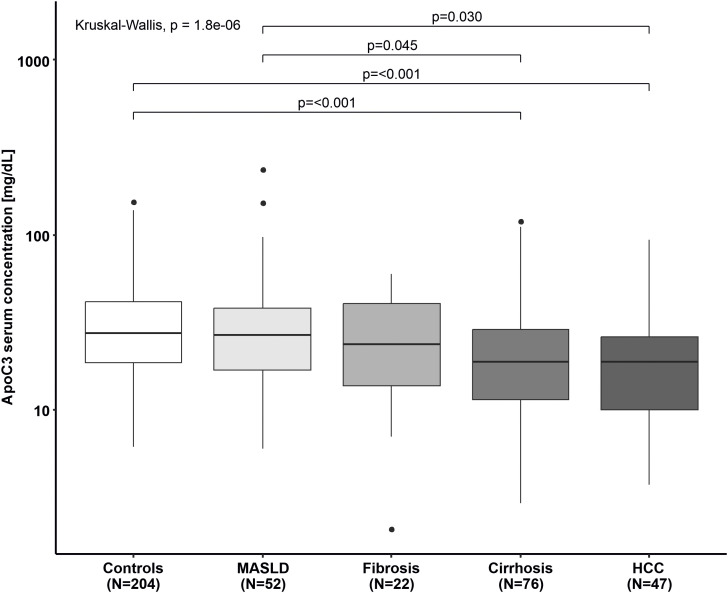
Apolipoprotein C3 (ApoC3) serum concentration in blood donor controls and in patients with MASLD across different stages of disease progression. Box plots display the median value (-) and the range of the data set, as indicated by the whiskers. Statistics: Kruskal-Wallis test (Dunn’s test post hoc) HCC: hepatocellular carcinoma, MASLD: metabolic dysfunction-associated steatotic liver disease.

Serum ApoC3 levels were significantly lower in the overall MASLD cohort compared to blood donor controls (median 20.1 mg/dL [range 2.8–235.0] vs. 27.4 mg/dL [6.1–154.7], p = 5.10x10^-06^). Within the MASLD cohort, patients with cirrhosis (18.7 mg/dL [2.9–119.3]) and those with cirrhosis with HCC (18.7 mg/dL [3.7–93.2]) had markedly lower ApoC3 concentrations than patients with non-fibrotic MASLD (26.6 mg/dL [6.0–235.0]) (p = 0.045 and p = 0.030, respectively). In addition, ApoC3 levels in patients with cirrhosis with and without HCC were significantly lower than those observed in the blood donor controls (cirrhosis: p = 7.61x10^-05^, cirrhosis and HCC: p = 0.0002). In contrast to the significant reductions observed in advanced disease stages, ApoC3 levels in patients with fibrosis did not differ significantly from those with non-fibrotic MASLD (23.8 mg/dL [2.1–59.9]; p = 1.000), or from controls (Fig 3).

To further disentangle the effect of malignancy, cirrhotic patients were additionally analyzed irrespective of HCC status. Patients with cirrhosis without HCC (n = 119) showed significantly lower ApoC3 levels compared to non-fibrotic MASLD (median 18.7 mg/dL [2.9–119.3] vs. 26.6 mg/dL [6.0–235.0], p = 0.013). In contrast, patients with HCC in the absence of cirrhosis (n = 4) exhibited a similar numerical decrease (median 16.8 mg/dL [8.7–93.2]) that did not reach statistical significance due to the small sample size (p = 1.000).

Among patients with cirrhosis, ApoC3 levels further declined with worsening liver function according to Child–Pugh class, with the lowest concentrations observed in class C (20.7 mg/dL [4.1–119.3] vs. 17.7 mg/dL [5.8–60.8] vs. 7.0 mg/dL [2.9–8.1]; p = 0.012) (S1 Fig).

**Fig 4:**
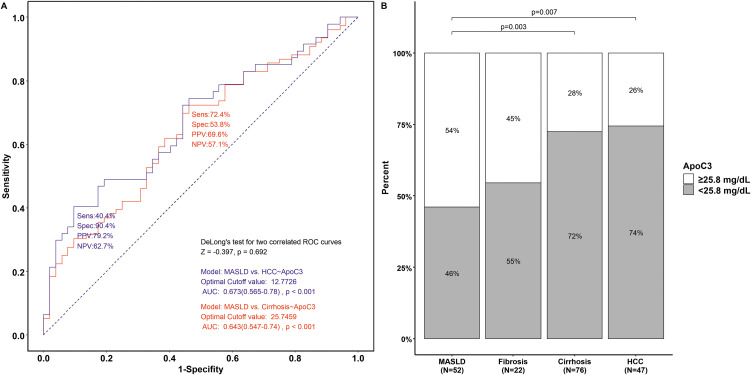
Diagnostic accuracy of ApoC3 in MASLD with cirrhosis with and without HCC. **A)** Receiver operating characteristics (ROC) curves of serum ApoC3 levels for the differentiation between patients with non-cirrhotic MASLD, cirrhosis with and without HCC. **B)** Application of the ApoC3 cut-off value of 25.8 mg/dL to differentiate between non-cirrhotic MASLD and patients with cirrhotic MASLD with and without HCC. AUC, area under the ROC; CI, confidence interval; HCC, hepatocellular carcinoma; MASLD, metabolic dysfunction-associated steatotic liver disease; NPV, negative predictive value; PPV, positive predictive value.

ROC analysis showed that ApoC3 levels were statistically associated with advanced liver disease, but the discriminative ability was modest (Fig 4). For cirrhosis, the AUC was 0.642 (95% CI: 0.546–0.738; p = 0.006), indicating limited sensitivity and specificity. The optimal cut-off of 25.8 mg/dL yielded 73% sensitivity, 54% specificity, a PPV of 70%, and an NPV of 57%. For HCC, the AUC was 0.673 (95% CI: 0.565–0.780; p = 0.003), with an optimal cut-off of 12.8 mg/dL achieving 40% sensitivity and 90% specificity (PPV: 79%, NPV: 63%). The ROC curves for cirrhosis with HCC did not differ significantly (p = 0.692).

Low ApoC3 levels (< 25.8 mg/dL) were found in 72% of patients with cirrhosis and 74% of patients with cirrhosis and HCC versus 46% of patients with non-fibrotic MASLD (p = 0.003, p = 0.007). Univariate analysis showed that ApoC3 < 25.8 mg/dL was significantly associated with cirrhosis (OR: 3.06, p = 0.003) and HCC (OR: 3.40, p = 0.005). In multivariate analysis adjusted for age, sex, and diabetes, the association remained significant for cirrhosis (adjusted OR: 3.39, p = 0.010, p adjusted = 0.04), but not for HCC (adjusted OR: 1.47, p = 0.633, p adjusted = 1.000) (S4 Table).

These cut-offs were derived in an exploratory manner and should be interpreted with caution given the cross-sectional study design. Overall, the ROC analyses suggest that ApoC3 has only a modest ability to discriminate advanced liver disease from non-fibrotic MASLD, and therefore should not be used as a standalone diagnostic marker. Rather, ApoC3 may provide complementary information when integrated into multi-parameter models including fibrosis scores, liver stiffness measurements, and metabolic risk factors.

### Association with cardiometabolic risk factors

**Fig 5:**
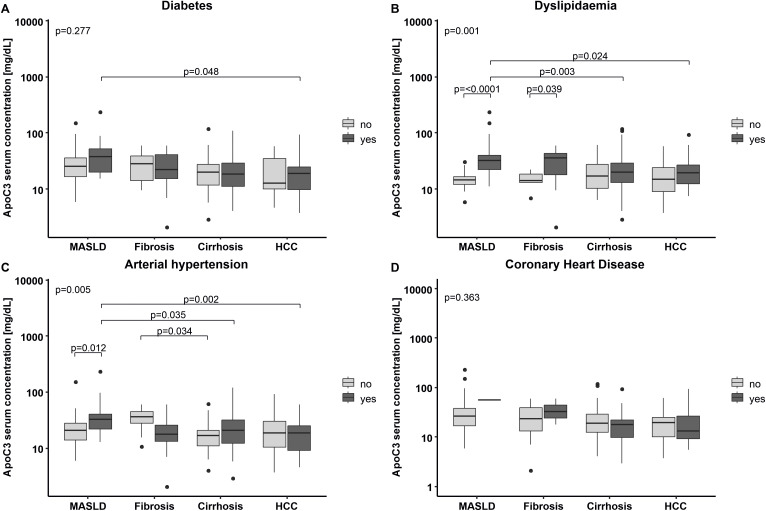
Serum apolipoprotein C3 (ApoC3) concentration in patients with MASLD across different stages of disease progression according to the presence of diabetes (A), dyslipidemia (B), arterial hypertension (C) and coronary heart disease (D). Box plots display the median value (-) and the range of the data set, as indicated by the whiskers. Statistics: Aligned rank transform (ART) ANOVA test (Dunn’s test post hoc) HCC: hepatocellular carcinoma, MASLD: metabolic dysfunction-associated steatotic liver disease.

The prevalence of type 2 diabetes increased with disease severity, highest in the cirrhosis and HCC group (77%), followed by cirrhosis (66%), fibrosis (50%), and non-fibrotic MASLD (19%). Among patients with diabetes, differences were observed between disease groups (Fig 5). Patients with MASLD and diabetes had significantly higher serum ApoC3 levels compared to patients with cirrhosis and HCC and diabetes (median 38.1 mg/dL [15.5–235] vs. 19.2 mg/dL [3.7–93.2], p = 0.048).

Patients with dyslipidemia showed significantly higher serum ApoC3 levels than patients without, seen in the group of MASLD and fibrosis equally (MASLD: median 32.0 mg/dL [11.2–235.0] vs 14.5 mg/dL [6.0–30.6], p = 0.0001; fibrosis: median 35.7 mg/dL [2.1–59.9] vs.14.3 mg/dL [7.0–22.0], p = 0.039). Furthermore, patients with MASLD and dyslipidemia had higher serum ApoC3 levels compared to patients with cirrhosis with and without HCC and dyslipidemia (cirrhosis: median 32.0 mg/dL [11.2–235.0] vs. 19.8 [2.9–119.0], p = 0.003; HCC: median 32.0 mg/dL [11.2–235.0] vs. 19.7 mg/dL [7.6–93.2], p = 0.024). Lipid-lowering therapy data were available for 94% of patients; 26% received treatment, mostly statins (85%).

With respect to arterial hypertension, patients within the MASLD group with hypertension exhibited significantly higher serum ApoC3 levels than those with normal blood pressure (median 32.4 mg/dL [12.9–235.0] vs. 20.8 mg/dL [6.0–153.0], p = 0.012). In addition, patients with MASLD and arterial hypertension had higher ApoC3 levels than patients with cirrhosis with and without HCC and arterial hypertension (cirrhosis: median 32.4 mg/dL [12.9–235.0] vs. 21.0 [2.9–119.0], p = 0.035; HCC: median 32.4 mg/dL [12.9–235.0] [11.2–235.0] vs. 18.7 mg/dL [4.7–60.9], p = 0.002).

No differences in serum ApoC3 levels were observed between the groups in patients with coronary heart disease.

### Serum ApoC3 concentrations and polygenic risk for MASLD progression

To evaluate the contribution of host genetics to MASLD progression, we analyzed common risk variants and calculated the polygenic risk score (PRS-5) based on five established loci [25]. A detailed description of genotype distributions and PRS-5 associations is provided in the Supplementary Material (S1 Fig, S5 Table).

In the context of lipid metabolism, we further examined the relationship between ApoC3 levels and genetic risk: ApoC3 levels showed a weak inverse correlation with the PRS-5 (r = –0.135, p = 0.007). Furthermore, two *ApoC3* single nucleotide polymorphisms (rs2854116, rs2854117) were genotyped (S5 Table). Although genotype frequencies did not differ significantly, ApoC3 levels tended to be lower in rs2854117 TT homozygotes, reaching significance in non-fibrotic MASLD (CC/CT vs TT: 18.8 vs. 27.8 mg/dL, p = 0.037) (see S2 Fig). These analyses were exploratory in nature.

## Discussion

Our study demonstrates a biphasic, stage-dependent pattern of ApoC3 in patients with MASLD. ApoC3 concentrations were elevated in early disease stages, particularly in the presence of metabolic and cardiometabolic disturbances, but declined in advanced stages, likely reflecting impaired hepatic synthetic function rather than metabolic improvement. These findings reinforce that MASLD is a systemic disorder in which biomarker interpretation depends on disease stage, metabolic context, and hepatic functional capacity.

Insulin resistance is a well-established pathophysiological driver of MASLD and is known to upregulate ApoC3 expression [13]. Our observations of higher ApoC3 levels in patients with early-stage MASLD and metabolic comorbidities are consistent with this regulatory mechanism, linking impaired insulin signaling to both hepatic lipid accumulation and systemic dyslipidemia. ApoC3 inhibits lipoprotein lipase, reducing the clearance of triglyceride-rich lipoproteins such as very low-density lipoproteins (VLDL), which may contribute to hepatic steatosis as well as atherogenic dyslipidemia, highlighting its dual role in liver and cardiovascular disease [8,10,12,26,27].

In line with prior literature [9–12,28,29], we observed the highest ApoC3 concentrations in patients with pronounced hepatic steatosis (S3) and high inflammatory activity (NAS ≥ 5). Together, these findings support a model in which ApoC3 reflects metabolic dysregulation in early MASLD while losing its discriminatory capacity in advanced stages due to declining liver function.

Our ROC analyses indicated that ApoC3 levels could distinguish advanced liver disease from non-fibrotic MASLD; however, the discriminative ability was modest (AUC for cirrhosis 0.642, for HCC 0.673). The optimal cut-offs derived in this exploratory analysis should be interpreted cautiously and do not imply immediate clinical applicability.

Histology-based analysis confirmed that ApoC3 levels were not significantly associated with fibrosis stage, and there was no difference between cirrhosis with or without HCC. This suggests that ApoC3 is more reflective of metabolic and inflammatory status than of fibrosis per se, and that declining ApoC3 in advanced disease likely represents hepatic insufficiency rather than protection from cardiometabolic risk.

Our exploratory polygenic risk score (PRS) analysis, derived from a relatively small cohort with overrepresentation of advanced disease stages should be considered hypothesis-generating. While informative regarding allele-specific regulation, the PRS provides limited additional insight for MASLD staging or risk stratification in this population. Genetic analyses revealed only a modest association of ApoC3 with the rs2854117 variant, suggesting that circulating ApoC3 is predominantly influenced by metabolic factors, insulin resistance, and hepatic function rather than fibrosis-related genetic predisposition.

Several limitations merit consideration. The retrospective, cross-sectional design precludes causal inference or longitudinal assessment of ApoC3 dynamics. Lipid-lowering therapies, particularly statins (26% of patients, 85% of these on statins), may confound ApoC3 levels indirectly via effects on hepatic lipid metabolism and insulin sensitivity. Direct ApoC3-targeting agents or fibrates were used infrequently, but their potential influence cannot be excluded.

In conclusion, our findings support a biphasic, stage-dependent role of ApoC3 in MASLD. Elevated ApoC3 levels in early disease reflect hepatic steatosis, inflammation, and systemic metabolic dysfunction, whereas declining levels in advanced MASLD likely mirror impaired hepatic synthetic function. ApoC3 links liver disease with cardiometabolic risk through its inhibition of lipoprotein lipase and modulation of triglyceride-rich lipoproteins. While not a standalone diagnostic biomarker, ApoC3 may provide complementary information when interpreted within the context of metabolic status, fibrosis, and cardiovascular risk. Future prospective interdisciplinary studies integrating longitudinal biomarker trajectories, proteoform-specific analyses, and cardiovascular outcomes are needed to clarify the clinical relevance of ApoC3 across the MASLD spectrum.

## Supporting information

S1 File Genetic risk and ApoC3 associations. (DOCX)

S1 Table Correlation of serum apolipoprotein C3 (ApoC3) concentration with laboratory parameters, non-invasive measures for steatosis and fibrosis and MASLD scores for non-invasive assessment of liver disease. Spearman’s correlation coefficient (r-value). (DOCX)

S2 Table Correlation of serum apolipoprotein C3 (ApoC3) concentration with laboratory parameters, non-invasive measures for steatosis and fibrosis and MASLD scores for non-invasive assessment of liver disease. Spearman’s correlation p-value. (DOCX)

S3 Table Characteristics of patients with and without liver biopsy. (DOCX)

S4 Table Association between low ApoC3 levels (<25.8 mg/dL) with the presence of liver cirrhosis or HCC in patients with MASLD. (DOCX)

S5 Table Genotype frequencies of patatin-like phospholipase domain-containing protein (PNPLA3), transmembrane 6 superfamily member 2 (TM6SFS), membrane bound O-acyltransferase domain containing 7 (MBOAT7), glucokinase regulator (GCKR), hydroxysteroid 17-beta-dehydrogenase 13 (HSD17B13) and apolipoprotein C3 (APOC3) and the polygenic risk (PRS-5) score in the study cohort groups. (DOCX)

S1 Fig Apolipoprotein C3 (ApoC3) serum concentration in patients with liver cirrhosis only and in patients with liver cirrhosis and HCC according to the Child Pugh classes. Box plots display the median value (-) and the range of the data set, as indicated by the whiskers. Statistics: Kruskal-Wallis test (Dunn’s test post hoc) HCC: hepatocellular carcinoma.
(TIFF)

S2 Fig Apolipoprotein C3 (ApoC3) serum concentration in A) blood donor controls and in patients and B) in patients with MASLD at different stages of disease progression according to the polygenic risk score (PRS-5) groups. Box plots display the median value (-) and the range of the data set, as indicated by the whiskers. Statistics: Aligned rank transform (ART) ANOVA test (Dunn’s test post hoc). HCC: hepatocellular carcinoma, MASLD: metabolic dysfunction-associated steatotic liver disease.
(TIF)

S3 Fig Apolipoprotein C3 (ApoC3) serum concentration in blood donor controls and in patients, and in patients with MASLD at different stages of disease progression according to the APOC3 polymorphisms rs2845116 (A-B) and rs2845117 (C-D). Box plots display the median value (-) and the range of the data set, as indicated by the whiskers. Statistics: Aligned rank transform (ART) ANOVA test (Dunn’s test post hoc). HCC: hepatocellular carcinoma, MASLD: metabolic dysfunction-associated steatotic liver disease.
(TIFF)

## Abbreviations

ALT: alanine aminotransferase
AP: alkaline phosphatase
ApoC3: apolipoprotein C3
ART: Aligned rank transform
AST: aspartate aminotransferase
AUC: area under the ROC curve
BMI: body mass index
CAP: controlled attenuation parameter
CHD: coronary heart disease
CI: confidence interval
CRP: C-reactive protein
CVD: cardiovascular disease
ELISA: enzyme-linked immunosorbent assay
FAST score: FibroScan-AST Score
FIB-4: fibrosis-4 score
GCKR: glucokinase regulator
GGT: gamma–glutamyl transpeptidase
HCC: hepatocellular carcinoma
HDL: high density lipoproteins
HSD17B3: hydroxysteroid 17-beta-dehydrogenase 13
ICH-GCP: International Council for Harmonisation-Good Clinical Practice
LDL: low-density lipoproteins
LSM: liver stiffness measurement
MASH: metabolic dysfunction-associated steatohepatitis
MASLD: metabolic dysfunction-associated steatotic liver disease
MBOAT7: membrane bound O-acyltransferase domain containing 7
METAVIR: meta-analysis of histological data in viral hepatitis
NAFLD: non-alcoholic fatty liver disease
NAS: NAFLD activity score
NFS: non-alcoholic fatty liver disease fibrosis score
NPV: negative predictive value
PNPLA3: patatin-like phospholipase domain-containing protein
PPV: positive predictive value
PRS-5: polygenic risk score
r: correlation coefficient
ROC: receiver operating characteristics
TG: triglyceride
TM6SF2: transmembrane 6 superfamily member 2
VCTE: vibration-controlled transient elastography
VLDL: very low-density lipoproteins
