# Supplementary material for: Stage-dependent dynamics of Apolipoprotein C3 across the spectrum of MASLD
Source: PLoS One. 2026 Jun 23;21(6):e0349666. doi: 10.1371/journal.pone.0349666 (PMC13289899; doi:10.1371/journal.pone.0349666)
Supplement: S1 File — (DOCX) [file pone.0349666.s001.docx]

**S1 – Genetic risk and ApoC3 associations**

**Genetic Risk Variants and PRS-5 Score**

Polymorphisms in PNPLA3, TM6SF2, MBOAT7, GCKR, and HSD17B13 were genotyped to assess genetic risk for MASLD progression. A polygenic risk score (PRS-5) was calculated based on the cumulative number of risk alleles (see Supplementary Table S1). Genotype distributions differed significantly between patients and controls for PNPLA3 and TM6SF2. The PNPLA3 GG risk variant was more prevalent in MASLD patients (21%) compared to controls (6%, p=4.65×10-06), whereas the favorable TM6SF2 CC genotype (69% vs. 92%, p = 3.29×10-09) and the protective HSD17B13 A allele (38% vs. 54%, p=0.007) were less common in patients. Consequently, PRS-5 scores were significantly elevated in patients (median 0.55 vs. 0.191, p=6.62×10-10).

Within the MASLD cohort, the PNPLA3 GG genotype was more frequent among patients with HCC than in non-cirrhotic MASLD (23% vs. 13%, p=0.044), while the TM6SF2 CC genotype was less frequent in HCC patients (55% vs. 83%, p=0.006). PRS-5 scores increased with disease severity. No significant differences were found for MBOAT7, GCKR, or HSD17B13 genotypes across subgroups.

**ApoC3 Associations with Genetic Risk**

ApoC3 serum levels were assessed in relation to genetic risk. While no significant correlations were observed between ApoC3 and individual risk variants, a weak inverse correlation was noted with PRS- 5 (r = –0.135, p=0.007). Stratification into low and high genetic risk groups (PRS-5 < 0.495 vs. ≥ 0.495) revealed no significant differences in ApoC3 levels across MASLD disease stages (see Supplementary S1 Fig).
