# Supplementary material for: Stage-dependent dynamics of Apolipoprotein C3 across the spectrum of MASLD
Source: PLoS One. 2026 Jun 23;21(6):e0349666. doi: 10.1371/journal.pone.0349666 (PMC13289899; doi:10.1371/journal.pone.0349666)
Supplement: S1 Table — (DOCX) [file pone.0349666.s002.docx]

**S1 Table: Correlation of serum apolipoprotein C3 (ApoC3) concentration with laboratory parameters, non-invasive measures for steatosis and fibrosis and MASLD scores for non-invasive assessment of liver disease. Spearman’s correlation coefficient (r-value).**

| **Parameter** | **APOC3** | **ALT** | **AST** | **AST/ALT ratio** | **AP** | **GGT** | **Bilirubin** | **Platelets** | **Albumin** | **Leuco-cytes** | **Choles-terol** | **LDL** | **HDL** | **Trigly-cerides** | **CRP** | **LSM** | **CAP** | **FIB-4 Score** | **FAST Score** | **NFS** |
| --- | --- | --- | --- | --- | --- | --- | --- | --- | --- | --- | --- | --- | --- | --- | --- | --- | --- | --- | --- | --- |
| **APOC3** | 1.000 | 0.098 | 0.024 | -0.117 | -0.162 | -0.010 | -0.314 | 0.263 | 0.291 | 0.174 | 0.300 | 0.289 | -0.053 | 0.485 | -0.177 | -0.193 | 0.274 | -0.201 | -0.093 | -0.199 |
| **ALT** | 0.098 | 1.000 | 0.635 | -0.659 | -0.010 | 0.231 | -0.024 | 0.290 | 0.294 | 0.119 | 0.227 | 0.202 | 0.030 | 0.191 | -0.141 | -0.160 | 0.282 | -0.293 | 0.279 | -0.445 |
| **AST** | 0.024 | 0.635 | 1.000 | 0.099 | 0.365 | 0.509 | 0.250 | 0.009 | -0.074 | 0.122 | 0.263 | 0.188 | -0.047 | 0.126 | 0.257 | 0.247 | 0.218 | 0.303 | 0.671 | 0.067 |
| **AST/ALT ratio** | -0.117 | -0.659 | 0.099 | 1.000 | 0.365 | 0.232 | 0.266 | -0.368 | -0.508 | -0.057 | -0.035 | -0.110 | -0.041 | -0.146 | 0.426 | 0.496 | -0.159 | 0.690 | 0.306 | 0.657 |
| **AP** | -0.162 | -0.010 | 0.365 | 0.365 | 1.000 | 0.552 | 0.218 | -0.182 | -0.470 | 0.130 | 0.100 | -0.006 | -0.041 | -0.005 | 0.512 | 0.291 | -0.007 | 0.384 | 0.308 | 0.345 |
| **GGT** | -0.010 | 0.231 | 0.509 | 0.232 | 0.552 | 1.000 | 0.110 | -0.151 | -0.233 | 0.059 | 0.178 | 0.018 | 0.060 | 0.093 | 0.391 | 0.368 | 0.168 | 0.317 | 0.479 | 0.185 |
| **Bilirubin** | -0.314 | -0.024 | 0.250 | 0.266 | 0.218 | 0.110 | 1.000 | -0.487 | -0.420 | -0.244 | -0.239 | -0.350 | -0.049 | -0.400 | 0.148 | 0.459 | -0.163 | 0.480 | 0.369 | 0.378 |
| **Platelets** | 0.263 | 0.290 | 0.009 | -0.368 | -0.182 | -0.151 | -0.487 | 1.000 | 0.536 | 0.545 | 0.326 | 0.389 | 0.101 | 0.308 | -0.225 | -0.630 | 0.251 | -0.825 | -0.394 | -0.773 |
| **Albumin** | 0.291 | 0.294 | -0.074 | -0.508 | -0.470 | -0.233 | -0.420 | 0.536 | 1.000 | 0.173 | 0.263 | 0.336 | 0.081 | 0.294 | -0.439 | -0.575 | 0.105 | -0.590 | -0.318 | -0.708 |
| **Leuco-cytes** | 0.174 | 0.119 | 0.122 | -0.057 | 0.130 | 0.059 | -0.244 | 0.545 | 0.173 | 1.000 | 0.176 | 0.170 | -0.038 | 0.279 | 0.163 | -0.139 | 0.341 | -0.378 | -0.016 | -0.276 |
| **Choles-terol** | 0.300 | 0.227 | 0.263 | -0.035 | 0.100 | 0.178 | -0.239 | 0.326 | 0.263 | 0.176 | 1.000 | 0.914 | 0.177 | 0.439 | 0.040 | -0.096 | 0.063 | -0.138 | 0.016 | -0.246 |
| **LDL** | 0.289 | 0.202 | 0.188 | -0.110 | -0.006 | 0.018 | -0.350 | 0.389 | 0.336 | 0.170 | 0.914 | 1.000 | 0.084 | 0.383 | -0.016 | -0.135 | 0.076 | -0.244 | -0.028 | -0.299 |
| **HDL** | -0.053 | 0.030 | -0.047 | -0.041 | -0.041 | 0.060 | -0.049 | 0.101 | 0.081 | -0.038 | 0.177 | 0.084 | 1.000 | -0.379 | -0.136 | -0.057 | -0.006 | -0.070 | 0.015 | -0.207 |
| **Trigly-cerides** | 0.485 | 0.191 | 0.126 | -0.146 | -0.005 | 0.093 | -0.400 | 0.308 | 0.294 | 0.279 | 0.439 | 0.383 | -0.379 | 1.000 | -0.082 | -0.132 | 0.302 | -0.194 | 0.018 | -0.129 |
| **CRP** | -0.177 | -0.141 | 0.257 | 0.426 | 0.512 | 0.391 | 0.148 | -0.225 | -0.439 | 0.163 | 0.040 | -0.016 | -0.136 | -0.082 | 1.000 | 0.373 | 0.185 | 0.376 | 0.370 | 0.409 |
| **LSM** | -0.193 | -0.160 | 0.247 | 0.496 | 0.291 | 0.368 | 0.459 | -0.630 | -0.575 | -0.139 | -0.096 | -0.135 | -0.057 | -0.132 | 0.373 | 1.000 | 0.049 | 0.722 | 0.772 | 0.661 |
| **CAP** | 0.274 | 0.282 | 0.218 | -0.159 | -0.007 | 0.168 | -0.163 | 0.251 | 0.105 | 0.341 | 0.063 | 0.076 | -0.006 | 0.302 | 0.185 | 0.049 | 1.000 | -0.172 | 0.411 | -0.105 |
| **FIB-4 Score** | -0.201 | -0.293 | 0.303 | 0.690 | 0.384 | 0.317 | 0.480 | -0.825 | -0.590 | -0.378 | -0.138 | -0.244 | -0.070 | -0.194 | 0.376 | 0.722 | -0.172 | 1.000 | 0.595 | 0.867 |
| **FAST Score** | -0.093 | 0.279 | 0.671 | 0.306 | 0.308 | 0.479 | 0.369 | -0.394 | -0.318 | -0.016 | 0.016 | -0.028 | 0.015 | 0.018 | 0.370 | 0.772 | 0.411 | 0.595 | 1.000 | 0.472 |
| **NFS** | -0.199 | -0.445 | 0.067 | 0.657 | 0.345 | 0.185 | 0.378 | -0.773 | -0.708 | -0.276 | -0.246 | -0.299 | -0.207 | -0.129 | 0.409 | 0.661 | -0.105 | 0.867 | 0.472 | 1.000 |

ALT: alanine transaminase, AP: alkaline phosphatase, AST: aspartate transaminase, CAP: controlled attenuation parameter, CRP: C-reactive protein, FAST: FibroScan-AST score, FIB-4: fibrosis-4 score, GGT: gamma-glutamyl transferase, HDL: high-density lipoprotein, LDL: low-density lipoprotein, LSM: liver stiffness measurement, NFS: non-alcoholic fatty liver disease fibrosis score.
