# Supplementary material for: Stage-dependent dynamics of Apolipoprotein C3 across the spectrum of MASLD
Source: PLoS One. 2026 Jun 23;21(6):e0349666. doi: 10.1371/journal.pone.0349666 (PMC13289899; doi:10.1371/journal.pone.0349666)
Supplement: S2 Table — (DOCX) [file pone.0349666.s003.docx]

**S2 Table: Correlation of serum apolipoprotein C3 (ApoC3) concentration with laboratory parameters, non-invasive measures for steatosis and fibrosis and MASLD scores for non-invasive assessment of liver disease. Spearman’s correlation p-value.**

| **Parameter** | **APOC3** | **ALT** | **AST** | **AST/ALT ratio** | **AP** | **GGT** | **Bilirubin** | **Platelets** | **Albumin** | **Leuco-cytes** | **Choles-terol** | **LDL** | **HDL** | **Trigly-cerides** | **CRP** | **LSM** | **CAP** | **FIB-4 Score** | **FAST Score** | **NFS** |
| --- | --- | --- | --- | --- | --- | --- | --- | --- | --- | --- | --- | --- | --- | --- | --- | --- | --- | --- | --- | --- |
| **APOC3** | 0 | 0.174 | 0.743 | 0.104 | 0.026 | 0.893 | 1.1E-05 | 0.0003 | 6.9E-05 | 0.017 | 0.0003 | 0.0004 | 0.523 | 7.4E-10 | 0.021 | 0.025 | 0.004 | 0.006 | 0.340 | 0.009 |
| **ALT** | 0.174 | 0 | 3.3E-23 | 2.0E-25 | 0.887 | 0.001 | 0.744 | 5.4E-05 | 5.9E-05 | 0.105 | 0.007 | 0.015 | 0.723 | 0.022 | 0.066 | 0.066 | 0.003 | 4.6E-05 | 0.003 | 8.8E-10 |
| **AST** | 0.743 | 3.3E-23 | 0 | 0.171 | 2.4E-07 | 5.8E-14 | 0.001 | 0.908 | 0.321 | 0.095 | 0.002 | 0.023 | 0.576 | 0.133 | 0.001 | 0.004 | 0.023 | 2.4E-05 | 1.9E-15 | 0.380 |
| **AST/ALT ratio** | 0.104 | 2.0E-25 | 0.171 | 0 | 2.4E-07 | 0.001 | 0.0002 | 2.0E-07 | 2.9E-13 | 0.439 | 0.680 | 0.186 | 0.628 | 0.081 | 6.1E-09 | 1.3E-09 | 0.099 | 7.0E-28 | 0.001 | 9.2E-23 |
| **AP** | 0.026 | 0.887 | 2.4E-07 | 2.4E-07 | 0 | 1.8E-16 | 0.003 | 0.013 | 2.7E-11 | 0.079 | 0.239 | 0.947 | 0.629 | 0.951 | 9.5E-13 | 0.001 | 0.941 | 6.7E-08 | 0.001 | 3.5E-06 |
| **GGT** | 0.893 | 0.001 | 5.8E-14 | 0.001 | 1.8E-16 | 0 | 0.134 | 0.038 | 0.002 | 0.420 | 0.034 | 0.827 | 0.476 | 0.269 | 1.4E-07 | 1.5E-05 | 0.084 | 9.6E-06 | 1.8E-07 | 0.015 |
| **Bilirubin** | 1.1E-05 | 0.744 | 0.001 | 0.0002 | 0.003 | 0.134 | 0 | 2.0E-12 | 4.4E-09 | 0.001 | 0.005 | 1.8E-05 | 0.563 | 7.9E-07 | 0.054 | 4.4E-08 | 0.096 | 5.3E-12 | 9.8E-05 | 3.6E-07 |
| **Platelets** | 0.0003 | 5.4E-05 | 0.908 | 2.0E-07 | 0.013 | 0.038 | 2.0E-12 | 0 | 9.5E-15 | 6.3E-16 | 8.6E-05 | 1.4E-06 | 0.228 | 0.0002 | 0.003 | 1.0E-15 | 0.010 | 6.1E-48 | 3.2E-05 | 1.3E-35 |
| **Albumin** | 6.9E-05 | 5.9E-05 | 0.321 | 2.9E-13 | 2.7E-11 | 0.002 | 4.4E-09 | 9.5E-15 | 0 | 0.020 | 0.002 | 4.4E-05 | 0.340 | 0.0004 | 2.4E-09 | 3.0E-12 | 0.292 | 3.6E-18 | 0.001 | 1.3E-27 |
| **Leuco-cytes** | 0.017 | 0.105 | 0.095 | 0.439 | 0.079 | 0.420 | 0.001 | 6.3E-16 | 0.020 | 0 | 0.037 | 0.042 | 0.647 | 0.001 | 0.034 | 0.115 | 0.0004 | 9.9E-08 | 0.871 | 0.000 |
| **Choles-terol** | 0.0003 | 0.007 | 0.002 | 0.680 | 0.239 | 0.034 | 0.005 | 8.6E-05 | 0.002 | 0.037 | 0 | 1.6E-55 | 0.037 | 7.3E-08 | 0.646 | 0.311 | 0.541 | 0.105 | 0.877 | 0.005 |
| **LDL** | 0.0004 | 0.015 | 0.023 | 0.186 | 0.947 | 0.827 | 1.8E-05 | 1.4E-06 | 4.4E-05 | 0.042 | 1.6E-55 | 0 | 0.318 | 2.6E-06 | 0.852 | 0.155 | 0.459 | 0.003 | 0.784 | 0.000 |
| **HDL** | 0.523 | 0.723 | 0.576 | 0.628 | 0.629 | 0.476 | 0.563 | 0.228 | 0.340 | 0.647 | 0.037 | 0.318 | 0 | 3.2E-06 | 0.115 | 0.548 | 0.957 | 0.406 | 0.884 | 0.016 |
| **Trigly-cerides** | 7.4E-10 | 0.022 | 0.133 | 0.081 | 0.951 | 0.269 | 7.9E-07 | 0.0002 | 0.0004 | 0.001 | 7.3E-08 | 2.5E-06 | 3.2E-06 | 0 | 0.344 | 0.169 | 0.003 | 0.020 | 0.866 | 0.134 |
| **CRP** | 0.021 | 0.066 | 0.001 | 6.1E-09 | 9.5E-13 | 1.4E-07 | 0.054 | 0.003 | 2.4E-09 | 0.034 | 0.646 | 0.852 | 0.115 | 0.344 | 0 | 2.9E-05 | 0.068 | 4.7E-07 | 0.0002 | 7.4E-08 |
| **LSM** | 0.025 | 0.066 | 0.004 | 1.3E-09 | 0.001 | 1.5E-05 | 4.4E-08 | 1.0E-15 | 3.0E-12 | 0.115 | 0.311 | 0.155 | 0.548 | 0.169 | 2.9E-05 | 0 | 0.611 | 3.1E-22 | 1.5E-22 | 2.0E-16 |
| **CAP** | 0.004 | 0.003 | 0.023 | 0.099 | 0.941 | 0.084 | 0.096 | 0.010 | 0.292 | 0.0004 | 0.541 | 0.459 | 0.957 | 0.003 | 0.068 | 0.611 | 0 | 0.079 | 9.7E-06 | 0.303 |
| **FIB-4 Score** | 0.006 | 4.6E-05 | 2.4E-05 | 7.0E-28 | 6.7E-08 | 9.6E-06 | 5.28E-12 | 6.1E-48 | 3.6E-18 | 9.9E-08 | 0.105 | 0.003 | 0.406 | 0.020 | 4.7E-07 | 3.1E-22 | 0.079 | 0 | 2.3E-11 | 1.5E-53 |
| **FAST Score** | 0.340 | 0.003 | 1.9E-15 | 0.001 | 0.001 | 1.8E-07 | 9.8E-05 | 3.2E-05 | 0.001 | 0.871 | 0.877 | 0.784 | 0.884 | 0.866 | 0.0002 | 1.5E-22 | 9.7E-06 | 2.3E-11 | 0 | 9.0E-07 |
| **NFS** | 0.009 | 8.8E-10 | 0.380 | 9.2E-23 | 3.5E-06 | 0.015 | 3.6E-07 | 1.3E-35 | 1.3E-27 | 0.0002 | 0.005 | 0.0004 | 0.016 | 0.134 | 7.4E-08 | 2.0E-16 | 0.303 | 1.5E-53 | 9.0E-07 | 0 |

ALT: alanine transaminase, AP: alkaline phosphatase, AST: aspartate transaminase, CAP: controlled attenuation parameter, CRP: C-reactive protein, FAST: FibroScan-AST score, FIB-4: fibrosis-4 score, GGT: gamma-glutamyl transferase, HDL: high-density lipoprotein, LDL: low-density lipoprotein, LSM: liver stiffness measurement, NFS: non-alcoholic fatty liver disease fibrosis score.
