# Supplementary material for: Stage-dependent dynamics of Apolipoprotein C3 across the spectrum of MASLD
Source: PLoS One. 2026 Jun 23;21(6):e0349666. doi: 10.1371/journal.pone.0349666 (PMC13289899; doi:10.1371/journal.pone.0349666)
Supplement: S3 Table — (DOCX) [file pone.0349666.s004.docx]

**S3 Table. Characteristics of patients with and without liver biopsy**

| **Parameter** | **Patients with liver biopsy (n=53)** | **Patients without liver biopsy (n=144)** | **p-value** |
| --- | --- | --- | --- |
| Male sex | 35 (66%) | 73 (51%) | 0.075 |
| Age (years) | 60 (21-83) | 61 (26-79) | 0.997 |
| BMI (kg/m^2^) | 31.3 (18.4-64.3) | 29.7 (17.2-47.9) | 0.316 |
| Diabetes | 37 (70%) | 70 (49%) | 0.010 |
| Arterial hypertension | 35 (66%) | 86 (60%) | 0.510 |
| Dyslipidaemia | 26 (49%) | 93 (65%) | 0.051 |
| CHD | 5 (9%) | 25 (17%) | 0.189 |
| Previous heart attack | 2 (4%) | 5 (3%) | 1.000 |
| Previous stroke | 1 (2%) | 3 (2%) | 1.000 |
| Treatment with cholesterol- lowering drugs | 13 (25%) | 35 (24%) | 1.000 |
| CAP (db/m) | 320 (113-401) | 315 (100-400) | 0.641 |
| LSM (kPa) | 13.3 (3.6-63.0) | 9.3 (2.1-75.0) | 0.228 |
| FIB-4 Score | 2.77 (0.44-25.12) | 2.71 (0.24-12.84) | 0.951 |
| NFS | 0.47 (-6.22-6.37) | 0.36 (-4.57-7.47) | 0.680 |
| FAST-Score | 0.54 (0.03-0.94) | 0.67 (0.15-0.97) | 0.013 |
| ALT (U/L) | 48.5 (17.4-262.9) | 40.1 (3.8–204.8) | 0.017 |
| AST (U/L) | 52.7 (25.2–149.7) | 46.1 (13.8–308.4) | 0.111 |
| ALT/AST ratio | 1.0 (0.2-7.6) | 1.1 (0.4–8.4) | 0.154 |
| GGT (U/L) | 177.6 (26.4–1614) | 105.0 (16.2–1307.4) | 0.038 |
| AP (U/L) | 118.8 (38.4–552) | 101.4 (9.6–867.6) | 0.159 |
| Bilirubin (µmol/L) | 11.6 (4.6-517.2) | 11.8 (1.6-122.1) | 0.478 |
| Albumin (g/L) | 43.0 (30.4-51.6) | 44.4 (27.0-55.7) | 0.168 |
| Platelets (x10^9^/L) | 158 (44-508) | 188 (42-639) | 0.427 |
| Leucocytes (x10^9^/L) | 7.2 (3.1-16.9) | 6.4 (1.4-12.6) | 0.132 |
| Total cholesterol (mmol/L) | 5.35 (3.16-8.45) | 5.13 (0.76-6.42) | 0.436 |
| LDL (mmol/L) | 3.04 (1.47-6.55) | 3.18 (0.21-7.96) | 0.839 |
| HDL (mmol/L) | 1.36 (0.13-2.27) | 1.20 (0.11-3.01) | 0.323 |
| Triglycerides (mmol/L) | 1.69 (0.56-5.34) | 1.62 (0.47-6.34) | 0.734 |
| CRP (mg/L) | 4.48 (0.47-56.35) | 3.18 (0.30-57.33) | 0.225 |

Values are presented as frequency (%) or median (range). ALT: alanine aminotransferase, AP: alkaline phosphatase, AST: aspartate aminotransferase, BMI: body mass index, CAP: controlled attenuation parameter, CHD: coronary heart disease, CRP: C-reactive protein, FAST Score: FibroScan-AST Score, FIB-4: fibrosis-4 score, GGT: gamma–glutamyl transpeptitase, HDL: high density lipoprotein, LDL: low density lipoprotein, LSM: liver stiffness measurement, NFS: non-alcoholic fatty liver disease fibrosis score
