# Supplementary material for: Stage-dependent dynamics of Apolipoprotein C3 across the spectrum of MASLD
Source: PLoS One. 2026 Jun 23;21(6):e0349666. doi: 10.1371/journal.pone.0349666 (PMC13289899; doi:10.1371/journal.pone.0349666)
Supplement: S4 Table — (DOCX) [file pone.0349666.s005.docx]

**S4 Table: Association between low ApoC3 levels (<25.8 mg/dL) with the presence of liver cirrhosis or HCC in patients with MASLD**

|  | Liver cirrhosis | | | | | HCC | | | | |
| --- | --- | --- | --- | --- | --- | --- | --- | --- | --- | --- |
|  | **Univariate analysis** | | **Multivariate analysis** | | | **Univariate analysis** | | **Multivariate analysis** | | |
| Parameter | **OR (95% CI)** | **p-value** | **OR (95% CI)** | **p-value** | **adj. p-value*** | **OR (95% CI)** | **p-value** | **OR (95% CI)** | **p-value** | **adj. p-value*** |
| Age (years) | 1.11 (1.06-1.15) | 2.13x10^-06^ | 1.09 (1.04-1.14) | 0.0002 | 0.008 | 1.29 (1.16-1.42) | 1.46x10^-06^ | 1.26 (1.12-1.42) | 0.0001 | 0.0004 |
| Males | 1.52 (0.74-3.11) | 0.254 | 0.62 (0.24-1.55) | 0.307 | 1.000 | 10.93 (3.93-30.40) | 4.57x10^-06^ | 12.88 (2.37-70.00) | 0.003 | 0.012 |
| Diabetes | 8.08 (3.50-18.65) | 9.93x10^-07^ | 5.86 (2.25-5.27) | 0.0003 | 0.0012 | 13.75 (5-24-36-09) | 1.03x10^-07^ | 6.01 (1.28-28.36) | 0.023 | 0.092 |
| ApoC3 <25.8 mg/dL | **3.06 (1.46-6.42)** | **0.003** | **3.39 (1.34-8.56)** | **0.010** | **0.040** | **3.04 (1.45-7.98)** | **0.005** | 1.47 (0.30-7.14) | 0.633 | 1.000 |

*Adjusted p-value after Bonferroni correction, CI: confidence interval, HCC: hepatocellular carcinoma, MASLD: metabolic dysfunction-associated steatotic liver disease, OR: Odds ratio
