# Supplementary material for: Stage-dependent dynamics of Apolipoprotein C3 across the spectrum of MASLD
Source: PLoS One. 2026 Jun 23;21(6):e0349666. doi: 10.1371/journal.pone.0349666 (PMC13289899; doi:10.1371/journal.pone.0349666)
Supplement: S5 Table — (DOCX) [file pone.0349666.s006.docx]

**S5 Table. Genotype frequencies of patatin-like phospholipase domain-containing protein (PNPLA3), transmembrane 6 superfamily member 2 (TM6SFS), membrane bound O-acyltransferase domain containing 7 (MBOAT7), glucokinase regulator (GCKR), hydroxysteroid 17-beta-dehydrogenase 13 (HSD17B13) and apolipoprotein C3 (APOC3) and the polygenic risk (PRS-5) score in the study cohort groups.**

| **Polymorphism** | **MASLD (n=52)** | **Fibrosis (n=22)** | **Cirrhosis (n=76)** | **HCC (n=47)** | **Controls (n=204)** | **MASLD vs.**  **fibrosis*** | **MASLD vs.**  **cirrhosis*** | **MASLD vs.**  **HCC*** | **cirrhosis vs. HCC*** |
| --- | --- | --- | --- | --- | --- | --- | --- | --- | --- |
| *PNPLA3* rs738409 |  |  |  |  |  |  |  |  |  |
| CC | 27 (52%) | 10 (46%) | 27 (36%) | 13 (28%) | 118 (58%) |  |  |  |  |
| CG | 18 (35%) | 8 (36%) | 29 (38%) | 23 (49%) | 73 (36%) | 0.834 | 0.115 | 0.044 | 0.499 |
| GG | 7 (13%) | 4 (18%) | 20 (26%) | 11 (23%) | 13 (6%) |  |  |  |  |
| *TM6SF2* rs58542926 |  |  |  |  |  |  |  |  |  |
| CC | 43 (83%) | 14 (64%) | 52 (68%) | 26 (55%) | 188 (92%) |  |  |  |  |
| CT | 9 (17%) | 8 (36%) | 22 (29%) | 19 (40%) | 15 (7%) | 0.128 | 0.142 | 0.006 | 0.351 |
| TT | 0 | 0 | 2 (3%) | 2 (4%) | 1 (0.5%) |  |  |  |  |
| *MBOAT7* rs641738 |  |  |  |  |  |  |  |  |  |
| CC | 20 (38%) | 6 (27%) | 22 (29%) | 11 (23%) | 59 (29%) |  |  |  |  |
| CT | 25 (48%) | 11 (50%) | 34 (45%) | 29 (62%) | 107 (52%) | 0.460 | 0.197 | 0.261 | 0.174 |
| TT | 7 (13%) | 5 (23%) | 20 (26%) | 7 (15%) | 38 (19%) |  |  |  |  |
| *GCKR* rs1260326 |  |  |  |  |  |  |  |  |  |
| CC | 15 (29%) | 8 (36%) | 24 (32%) | 16 (34%) | 67 (33%) |  |  |  |  |
| CT | 27 (52%) | 7 (32%) | 42 (55%) | 24 (51%) | 99 (48%) | 0.277 | 0.663 | 0.797 | 1.000 |
| TT | 10 (19%) | 7 (32%) | 10 (13%) | 7 (15%) | 38 (19%) |  |  |  |  |
| *HSD17B13* rs72613567 |  |  |  |  |  |  |  |  |  |
| TT | 31 (60%) | 11 (50%) | 48 (63%) | 32 (68%) | 98 (48%) |  |  |  |  |
| AT | 17 (33%) | 7 (32%) | 19 (25%) | 10 (21%) | 89 (44%) | 0.346 | 0.589 | 0.451 | 0.886 |
| AA | 4 (8%) | 4 (18%) | 9 (12%) | 5 (11%) | 17 (8%) |  |  |  |  |
| PRS-5 | 0.208 (0-0.830) | 0.340 (0.024-0.731) | 0.420 (0-1.145) | 0.402 (0-1.143) | 0.191 (0-0.725) | 0.127 | 0.010 | 0.023 | 0.909 |
| < cut-off 0.495 | 39 (75%) | 14 (64%) | 42 (55%) | 25 (53%) | 181 (89%) |  |  |  |  |
| ≥ cut-off 0.495 | 13 (25%) | 8 (36%) | 341 (45%) | 22 (47%) | 23 (11%) | 0.400 | 0.026 | 0.035 | 0.854 |
| *APOC3* rs2854116 |  |  |  |  |  |  |  |  |  |
| TT | 15 (29%) | 11 (50%) | 27 (35%) | 14 (30%) | 77 (38%) |  |  |  |  |
| CT | 25 (48%) | 9 (41%) | 34 (45%) | 24 (51%) | 96 (47%) | 0.174 | 0.733 | 0.931 | 0.758 |
| CC | 12 (23%) | 2 (9%) | 15 (20%) | 9 (19%) | 31 (15%) |  |  |  |  |
| *APOC3* rs2854117 |  |  |  |  |  |  |  |  |  |
| CC | 23 (44%) | 14 (64%) | 41 (54%) | 22 (47%) | 108 (53%) |  |  |  |  |
| CT | 21 (40%) | 8 (36%) | 26 (34%) | 21 (45%) | 79 (39%) | 0.094 | 0.560 | 0.612 | 0.553 |
| TT | 8 (15%) | 0 | 9 (12%) | 4 (8%) | 17 (8%) |  |  |  |  |
| *p-value, PRS-5 score shown in median (range). HCC: hepatocellular carcinoma, MASLD: metabolic dysfunction-associated steatotic liver disease | | | | | | | | | |
