# Supplementary figures and images for: Stage-dependent dynamics of Apolipoprotein C3 across the spectrum of MASLD
Source: PLoS One. 2026 Jun 23;21(6):e0349666. doi: 10.1371/journal.pone.0349666 (PMC13289899; doi:10.1371/journal.pone.0349666)

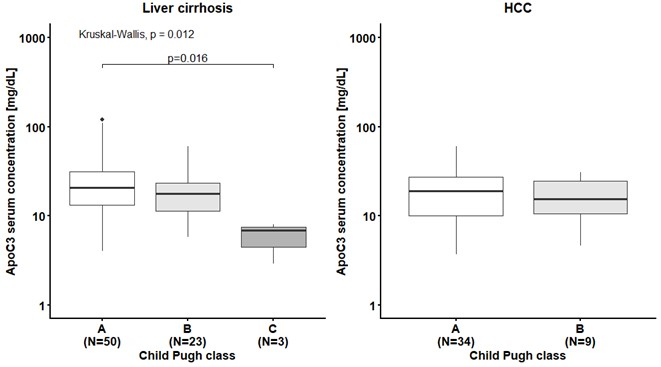

Supplement: S1 Fig — Box plots display the median value (-) and the range of the data set, as indicated by the whiskers. Statistics: Kruskal-Wallis test (Dunn’s test post hoc) HCC: hepatocellular carcinoma. (TIFF) [file pone.0349666.s007.tiff]

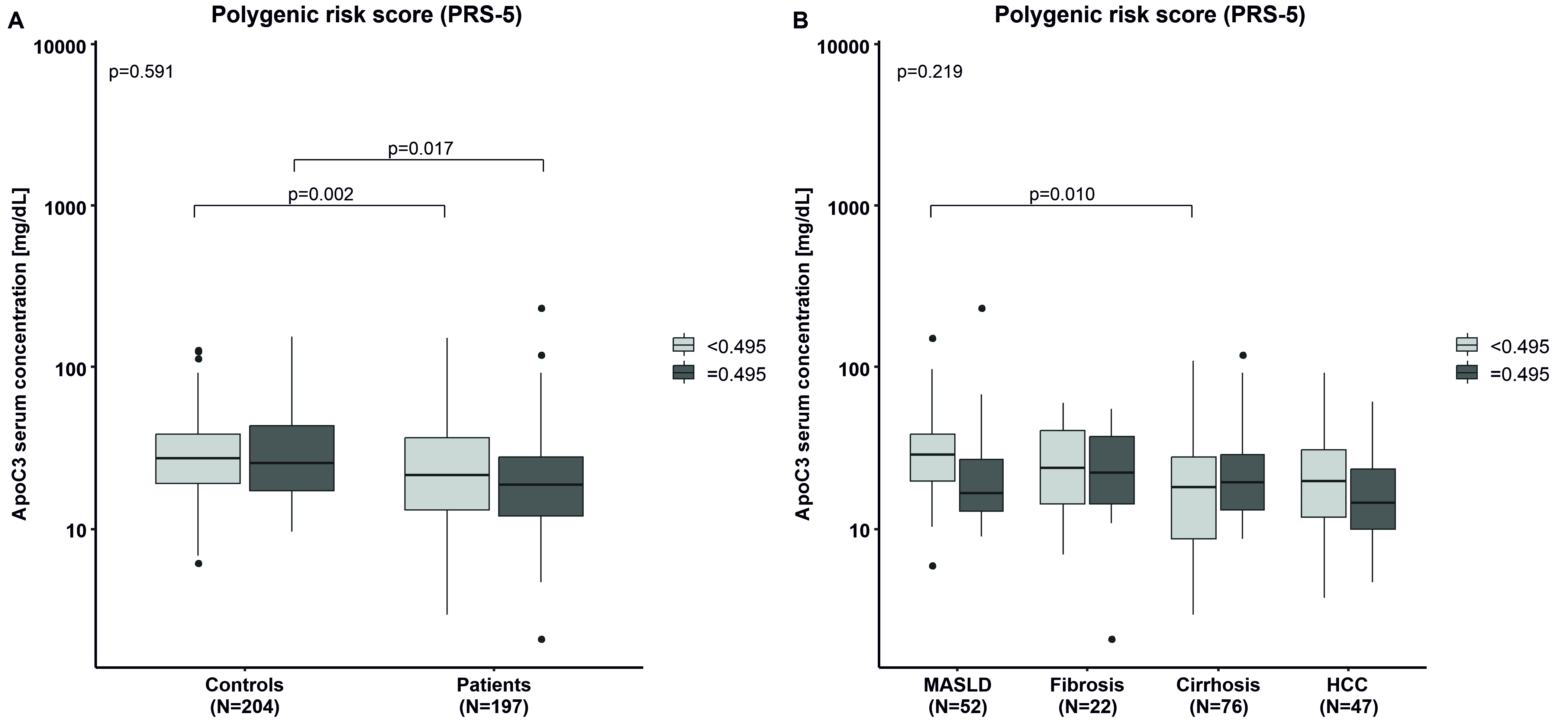

Supplement: S2 Fig — Box plots display the median value (-) and the range of the data set, as indicated by the whiskers. Statistics: Aligned rank transform (ART) ANOVA test (Dunn’s test post hoc). HCC: hepatocellular carcinoma, MASLD: metabolic dysfunction-associated steatotic liver disease. (TIF) [file pone.0349666.s008.tif]
